# The Role of Non-Cognate T Cell Stimulation during Intracellular Bacterial Infection

**DOI:** 10.3389/fimmu.2014.00319

**Published:** 2014-07-09

**Authors:** Stephen J. McSorley

**Affiliations:** ^1^Department of Anatomy, Physiology and Cell Biology, Center for Comparative Medicine, School of Veterinary Medicine, University of California Davis, Davis, CA, USA

**Keywords:** T cell, bacterial infections, protective immunity, IFN-gamma, *Salmonella*

## Abstract

Intra-macrophage bacterial infections cause significant morbidity and mortality in both the developed and developing world. Protective host immune responses to these infections initially requires the activation and expansion of pathogen-specific CD4 Th1 cells within lymphoid tissues and subsequent relocation of these effector cells to sites of infection. After entering infected tissues, the elicitation of Th1 bactericidal activity can be triggered by cognate or non-cognate signals that are delivered by locally infected antigen-presenting cells and innate cells. However, the contribution of non-cognate stimulation to the resolution of bacterial infection remains poorly understood, especially in the context of a Th1 response. Here, we review the current data on Th1 cell activation and expansion in mouse models of *Salmonella* and *Chlamydia* infection and discuss the potential role of non-cognate Th1 cell stimulation in these disease models. Greater understanding of this pathway of T cell activation may lead to the design of therapeutics or vaccines to combat intra-macrophage pathogens.

## Introduction

The mammalian immune system contains a variety of cell types that respond in a highly coordinated fashion to eradicate microbial pathogens. The different cell populations that mediate this host defense capability are conveniently assigned to innate or adaptive immune compartments depending a variety of factors, including the tempo of the effector function produced, the use of certain pathogen recognition receptors, and whether these cells have an inherent capacity to confer immune memory. Innate immune responses typically invoke an immediate effector response, make use of germ-line encoded receptors with a restricted capacity for pathogen recognition, and lack the ability to confer a stronger response to secondary infection (1). In contrast, adaptive immune responses require a period of maturation before effector functions are elaborated, utilize complex, rearranged receptors that allow a wider range of specificities, and confer a modified host response to re-infection (2). This general compartmentalization of cells into innate and adaptive arms of the immune system is useful since it provides a conceptual framework that reduces complexity in understanding the dynamics of host–pathogen interactions. However, as might be expected, this model is an oversimplification and some cells of the innate immune system can display characteristics of the adaptive response, and vice versa (3–6). In this review, we will discuss the capacity of adaptive Th1 cells to elaborate effector functions in response to innate stimuli and thus under these conditions appear to function as a component of the innate immune response. The ability of these expanded effector lymphocytes to blur the lines between innate and adaptive immunity may be a critical component of protective immunity to *Salmonella, Chlamydia*, and other intracellular bacteria.

## Global Impact of *Salmonella* and *Chlamydia* Infections

*Salmonella* can cause different clinical diseases in a human host, depending upon the genome of the infecting *Salmonella* serovar and the immune competence of the infected host (7, 8). Typhoid fever is caused by human transmission of *Salmonella enterica* serovar Typhi or serovar Paratyphi and this disease remains prevalent in parts of Africa and Asia (9). Current estimates suggest that typhoid causes 217,000 deaths globally every year, the impact of which is felt predominantly in geographical regions with limited access to clean water or basic sanitation infrastructure (10). Although typhoidal serovars enter the human host via the intestine, much of the *in vivo* bacterial replication occurs in the systemic tissues of the liver, spleen, and bone marrow. In contrast, many other *Salmonella* serovars can cause local gastro-intestinal infections that are often self-limiting but are a major cause of food-borne infection in the US and other developed nations (11, 12). Thus, *Salmonella* infection has a global footprint and largely affects developed and developing nations with different patterns of systemic or localized disease. A third disease caused by *Salmonella* has emerged in sub-Saharan Africa and primarily affects patients with an immature or compromised immune system, either due to age, co-infection, or nutritional status (13, 14). These *Salmonella* infections can be systemic and are caused by non-typhoidal serovars and therefore this disease is collectively referred to as invasive non-typhoidal Salmonellosis (NTS). While vaccines are currently available for typhoid, these are not widely used in typhoid endemic areas due to concerns about efficacy, safety, or cost (8). The development of improved vaccines for typhoid and NTS therefore remains a priority. Greater understanding of host protective immune mechanisms during *Salmonella* infection will be required in order to meet this important goal.

While *Salmonella* is a facultative intracellular pathogen that can grow inside and outside host cells, *Chlamydia* is an obligate intracellular organism and is only metabolically active within host cells (15). *Chlamydia trachomatis* causes a sexually transmitted infection in humans that is now the most common notifiable disease in the US (16). The 1.4 million *Chlamydia* cases reported in 2011 represent an 8% increase over 2010 and is the largest number of cases ever reported to the Centers for Disease Control (CDC) for any single condition (16). Overall, the CDC reports an 8.3% positivity rate among young women screened at family planning clinics, making *Chlamydia* one of the most prevalent bacterial infections in the US (17). Although most *Chlamydia* infections are initially asymptomatic, they cause serious pelvic inflammatory disease (PID) in 5–15% of untreated female patients (18, 19). Approximately one in six women who develop PID become infertile, while many others develop chronic pelvic pain, ectopic pregnancy, and if exposed to HIV, *Chlamydia*-infected women are five times more likely to acquire the virus (18–20). Thus, *Chlamydia* infection represents a growing healthcare problem in the US and greater understanding of protective immunity in the female reproductive tract will be required to develop an effective vaccine.

## Role of CD4 Th1 Cells in Protective Immunity to *Salmonella* and *Chlamydia*

Given the location of *Chlamydia* infection in the reproductive tract and *Salmonella* infection in the intestine, the immune response to these infections will undoubtedly contain unique tissue-specific components. However, in both mouse models of *Salmonella* and *Chlamydia* infection, pathogen-specific CD4 Th1 cells have been found to be essential for successful resolution of primary infection (21, 22). In the *Salmonella* model, oral infection of C57BL/6 mice with attenuated bacteria generates a systemic infection that eventually resolves over a period of several weeks (23). The ability to resolve this infection is absent in mice lacking MHC class-II-restricted T cells, IFN-γ, or the Th1 transcription factor T-bet (24, 25). Furthermore, successful resolution of *Salmonella* infection correlates with the expansion of *Salmonella*-specific Th1 cells in systemic tissues (23, 26).

Genital inoculation of C57BL/6 mice with *Chlamydia muridarum* generates a self-limiting ascending infection of the upper reproductive tract (21). Similar to *Salmonella* infection, the resolution of primary *C. muridarum* infection requires the presence of MHC class-II restricted T cells and IFN-γ (27). The *Chlamydia*-specific T cell response has been visualized using antigen-specific reagents and the predominant T helper subset detected in draining lymph nodes and spleen consists of a Th1 population that expresses T-bet and secretes IFN-γ (28, 29). In both infection models, the contribution of CD8 T cells and B cells in resolving primary infection is thought to be limited (27, 30–33), although recent data suggest a requirement for B cells in preventing bacterial dissemination to systemic tissues following *Chlamydia* genital challenge (28). It is not yet clear whether this implies a requirement for B cells in antigen presentation to CD4 T cells or simply a requirement for early antibody production.

Secondary responses to *Salmonella* and *Chlamydia* infection have also been examined and the data suggest a wider range of lymphocyte responses that can contribute to bacterial clearance (21, 34). Despite the fact that *Salmonella* and *Chlamydia* replicate intracellularly in an infected host, B cells and antibody can contribute to the resolution of secondary infection (30–32, 35, 36). A role for B cells is evident in experiments examining acquired immunity in B cell-deficient mice or by examining the protective immunity mediated by the transfer of immune serum (31, 32, 36–38). Similarly, CD8 T cells have been reported to contribute to secondary protection against both *Salmonella* and *Chlamydia* (24, 27, 39), although a recent report examining *Salmonella* infection of MHC class-I, perforin-, and granzyme-deficient mice did not detect an impaired protective response to secondary infection (33). Despite the expanded contribution of antibody and CD8 T cells in secondary protective immunity, CD4 Th1 cells are still thought to be the primary cell type involved in the resolution of secondary infection (21, 22). Thus, the development of pathogen-specific CD4 Th1 cells is essential for the development of protective immunity in mouse models of *Salmonella* and *Chlamydia* infection.

## Cognate Signals Driving T Cell Activation and Reactivation

Naïve pathogen-specific CD4 T cells are activated in secondary lymphoid tissues by dendritic cells expressing CD80/86 and displaying microbial peptides on surface MHC class-II (40). In both *Salmonella* and *Chlamydia* infection models, TCR transgenic mice and MHC class-II tetramers have been used to visualize naïve T cell activation, expansion, and acquisition of effector functions *in vivo* (28, 41–43). Initial T cell expansion occurs in the Peyer’s patch and mesenteric lymph nodes after oral infection with *Salmonella* (41, 44). However, systemic expansion of CD4 T cells can also occur in the spleen and recent evidence suggests that these mucosal and systemic responses are functionally and antigenically distinct (43). Thus, while flagellin-specific CD4 Th17 cells expand in the intestine of *Salmonella*-infected mice, CD4 Th1 cells specific for components of the *Salmonella* Pathogenicity Island 2 (SPI2) Type III Secretion System were expanded in the spleen (43). Genital infection of mice with *C. muridarum* initially drives *Chlamydia*-specific T cell expansion in the draining ileac lymph, before systemic expansion occurs in the spleen (28). Unlike *Salmonella* infection, the antigenic targets of the CD4 response appear to be similar in mucosal and systemic locations and Th1 cells were primarily detected both locally and systemically. The most prominent feature of the immune response in both *Salmonella* and *Chlamydia* infection models is that a large pool of expanded pathogen-specific Th1 cells is generated. The activation and clonal expansion of *Salmonella*-specific T cells is strictly dependent on cognate stimulation since flagellin-specific T cells remain unactivated after infection of mice with flagellin-deficient *Salmonella* (41, 44). Thus, Th1 cells arise from a relatively infrequent pool of naïve pathogen-specific T cells in response to cognate (TCR-dependent) signals that are delivered in lymphoid tissues and these signals eventually lead to clonal expansion and effector development.

## Non-Cognate Activation of Effector T Cells

**Figure 1:**
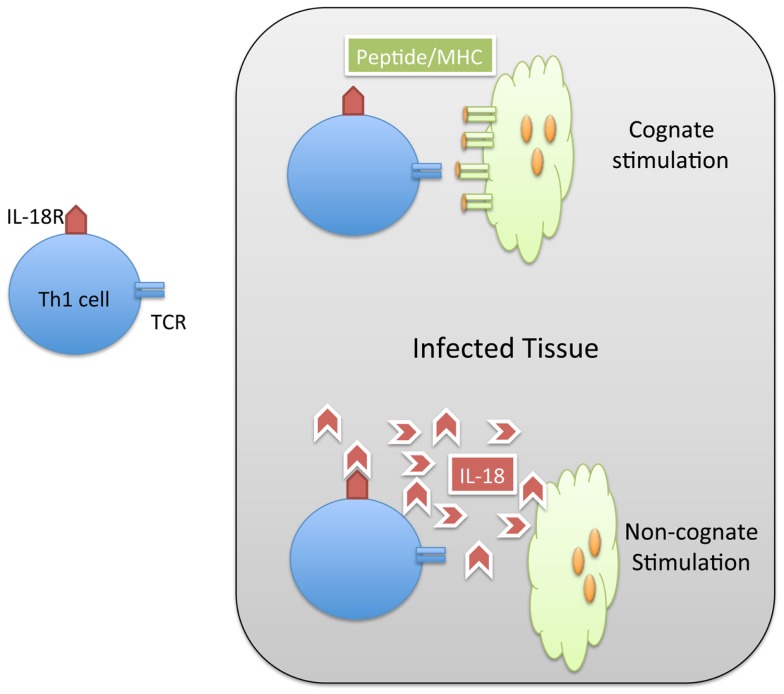
**Th1 cells can be activated by cognate and non-cognate stimuli in infected tissue**. Naïve CD4 T cells are activated in lymphoid tissues to generate Th1 cells specific for *Salmonella*. These *Salmonella*-specific T cells can enter infected tissue and be stimulated by cognate (MHC/peptide) stimuli (TOP) or non-cognate (IL-18) stimuli. In both cases, the result of this stimulation is the production of IFN-gamma and resolution of the infection.

When Th1 cells relocate to an infected non-lymphoid tissue, they can produce IFN-γ locally in order to restrain intracellular bacterial replication (45). The stimulatory signals required to elicit local IFN-γ from effector T cells in tissues could potentially involve cognate stimulation via peptide/MHC complexes on the surface of infected cells or resident dendritic cells (Figure 1). However, many intracellular pathogens have evolved strategies that prevent MHC presentation of microbial peptides or down-regulate surface MHC expression on infected cells (46, 47). While down-regulation of MHC class-I is often discussed as a viral evasion strategy (48), *Salmonella* have also been reported to reduce expression of MHC class-II of antigen-presenting cells (49). Thus, in the absence of cognate ligands, Th1 cells may simply recognize inflammatory cues such as cytokines and TLR ligands in infected tissues to secrete IFN-γ (Figure 1). However, the relative contribution of cognate versus non-cognate signals in the eradication of intracellular pathogens is not fully understood.

Effector CD4 T cells that have relocated to non-lymphoid tissues retain the ability to respond to cognate signals in that location. Indeed, in a non-infectious model system, antibody that effectively blocked the peptide/MHC complex reduced the ability of CD4 T cells to produce effector cytokines (50). Similarly, recent experiments with bone marrow chimeras containing MHC class-II-deficient and MHC class-II sufficient myeloid cells demonstrated an increased burden of *M. tuberculosis* in host cells lacking MHC class-II (51). These data support the idea that Th1 cells scan infected tissues and can respond to local cognate signals to produce cytokines. However, the ability to respond to cognate signals may not always be required for the elaboration of effector functions. Studies of CD8 T cell effector function have demonstrated that expanded pathogen-specific T cells can secrete IFN-γ in response to a variety of inflammatory cytokines including IL-12, IL-18, and IL-15 (52–54). In a similar manner, CD4 cells have been shown to produce cytokines after direct ligation of surface TLRs by microbial products (3). Thus, non-cognate stimulation of Th1 cells could potentially be a major contributing factor to bacterial clearance from tissues during intracellular infections.

In a mouse model of *Salmonella* infection, a large proportion of CD4 T cells can be rapidly induced to secrete IFN-γ following intravenous injection of heat-killed bacteria (23). It was initially assumed that this complex mixture of bacterial antigens was able to efficiently activate *Salmonella*-specific Th1 cells via cognate signals delivered after antigen presentation of heat-killed bacteria. However, it was subsequently demonstrated that this large response from Th1 cells could also be induced following the injection of TLR ligands and more importantly also occurred in the absence of host MHC class-II (55, 56). Recently, this response was shown to be due to the induction of IL-18 and IL-33 in response to both TLR and inflammasome stimulation (57). The primary inflammasome components involved in recognition of *Salmonella* infection are NLRC4 and NLRP3 (58). Although NLRC4 can be activated in response to flagellin, bacteria that lacked flagellin expression were still able to induce non-cognate T cell activation suggesting that other components also participate in this response. Overall, these data suggest that inflammasome activation combines with TLR ligation to induce IL-18 and IL-33 production and that these cytokines drive T cell stimulation. Indeed, optimal IFN-γ production required T cell expression of IL-18R and IL-33R and mice containing a T cell-specific deficiency in Myd88 were less able to control the growth of *Salmonella* (57). A very similar pathway of non-cognate T cell activation has been reported following the injection of bacterial flagellin, although activation of CD8 T cells in this case was thought to require direct flagellin recognition by NLRC4 expressed by dendritic cells (59). Together, these data suggest that, during *Salmonella* infection, non-cognate signals may be vitally important for driving CD4 Th1 and CD8 T cells to produce IFN-γ and that mice lacking these particular pathways may be unable to generate an effective adaptive response. Interestingly, a similar non-cognate response was detected from Th1 cells in *Chlamydia*-infected mice (57), suggesting that non-cognate activation of CD4 T cells may be a common feature of the host immunity to intracellular bacteria. Future experiments examining other intracellular pathogens will be important to determine how ubiquitous this pathway is for eliciting protective Th1 responses to microbial pathogens. However, the finding that clearance of *M. tuberculosis* from individual myeloid cells requires direct cognate stimulation implies that an appropriate balance of cognate and non-cognate signals in infected tissues will be important for Th1 responses to different intracellular pathogens (51). Indeed, it is possible that cognate and non-cognate signals are each responsible for Th1 cytokine production at different stages of the host response, in different anatomical locations, or simply depending on the overall bacterial load within an infected tissue.

## Contribution of Non-Cognate T Cell Activation to Pathogen Clearance

The non-cognate elicitation of an effector response from expanded T cells may be required to specifically deal with pathogens that are able to alter host MHC expression or affect the presentation of microbial peptides in infected tissues. Any Th1 cell that enters an infected tissue would therefore retain some capacity to produce IFN-γ in response to local inflammation. Indeed, it has been shown that IFN-γ produced locally can induce iNOS expression from locally infected macrophages, even an individual macrophage happens to lack expression of MHC class-II (45). Thus, there is a degree of non-specificity in the function of Th1 cells within infected tissues. The ability of these same T cells to respond to non-cognate signals may simply further decrease the activation threshold for eliciting bactericidal response. Although it has not been directly examined *in vivo*, the contribution of non-cognate Th1 cell stimulation may be directly related to the overall pathogen burden in the infected tissue. Thus, if the overall tissue burden is low, then PAMP-elicited cytokines such as IL-18 and IL-33 would also be expected to be at low concentrations, leaving Th1 cells to seek out cognate stimulation and thus constraining T cell activation to a very localized radius around the few infected cells in the tissue. In contrast, if a Th1 cell encounters high concentrations of inflammatory cytokines, the threshold for T cell stimulation would effectively be lowered, allowing immediate and widespread production of IFN-γ. Such a lower threshold of activation may be particularly important when an infected host is combating a rapidly dividing or rapidly spreading pathogen such as *Salmonella*, but conversely may be less important for immunity to a slow growing pathogen such as *M. tuberculosis*.

Another potential role for non-cognate T cell activation could occur in situations of bacterial co-infection. Indeed, a role for non-cognate T cell activation in driving pathology has been examined in the context of influenza and bacterial co-infections (60). In this case, an expanded pool of virus-specific CD8 T cells could be rapidly activated to produce harmful pathology in response to inflammatory cytokines elicited by bacterial infection. Conversely, persistent viral stimulation of macrophages can sometimes provide protection against some intracellular bacterial infections (61). In the case of Th1 cells, a pathway of non-cognate activation could be a primary driver of protective immunity during a co-infection. For example, if an individual is infected with an intracellular pathogen and therefore has invested in the expansion and functional maturation of a pool of Th1 cells, the simultaneous encounter with an unrelated secondary infection may well recruit and activate these Th1 cells in a non-cognate manner. Indeed, the original discovery of macrophage activation was surprising because the efferent phase of the adaptive response involved a relatively non-specific mechanism and was demonstrated using a co-infection model where *Brucella* infection prevented productive infection with *Listeria*. However, a role for non-cognate T cell activation in the elicitation of protective immunity during co-infections has not yet been described. Overall, it seems most likely that non-cognate mechanisms of Th1 cell activation could have evolved to help the host combat bacterial evasions of host immunity, superior bacterial cell division, or co-infections. Future research in this area is required to examine each of these possibilities.

## Conclusion

Naïve CD4 T cells are activated by cognate signals leading to the expansion of an effector pool of pathogen-specific T cells that can migrate to infected tissues and deliver local anti-microbial effects. Recent data have demonstrated that Th1 cells can be activated within infected tissues in response to cognate and/or non-cognate signals that arise from TLR and inflammasome activation. Thus, although the adaptive response is regulated by highly specific antigen-specific surface receptors, an expanded pool of effector cells retains the ability to respond immediately to inflammatory cues that are normally associated with the innate arm of the immune system. This functional capability reinforces our growing understanding that innate and adaptive immune systems are not completely separate entities but instead work in a coordinated fashion to resolve infection with microbial pathogens. This ability of expanded effector lymphocytes to blur the lines between innate and adaptive immunity may be a critical component of protective immunity to *Salmonella, Chlamydia*, and other intracellular bacteria.

## Conflict of Interest Statement

The author declares that the research was conducted in the absence of any commercial or financial relationships that could be construed as a potential conflict of interest.
